# Artocarpin, an isoprenyl flavonoid, induces p53-dependent or independent apoptosis via ROS-mediated MAPKs and Akt activation in non-small cell lung cancer cells

**DOI:** 10.18632/oncotarget.16058

**Published:** 2017-03-09

**Authors:** Ming-Horng Tsai, Ju-Fang Liu, Yao-Chang Chiang, Stephen Chu-Sung Hu, Lee-Fen Hsu, Yu-Ching Lin, Zih-Chan Lin, Hui-Chun Lee, Mei-Chuan Chen, Chieh-Liang Huang, Chiang-Wen Lee

**Affiliations:** ^1^ Department of Pediatrics, Division of Neonatology and Pediatric Hematology/Oncology, Chang Gung Memorial Hospital, Yunlin, Taiwan; ^2^ Central Laboratory, Shin-Kong Wu Ho-Su Memorial Hospital, Taipei, Taiwan; ^3^ Center for Drug Abuse and Addiction, China Medical University Hospital, China Medical University, Taichung, Taiwan; ^4^ Department of Nursing, Division of Basic Medical Sciences, Chang Gung University of Science and Technology, Chia-Yi, Taiwan; ^5^ Department of Dermatology, College of Medicine, Kaohsiung Medical University, Kaohsiung, Taiwan; ^6^ Department of Dermatology, Kaohsiung Medical University Hospital, Kaohsiung, Taiwan; ^7^ Department of Respiratory Care, Chang Gung University of Science and Technology, Chiayi Campus, Chiayi, Taiwan; ^8^ Division of Pulmonary and Critical Care Medicine, Chang Gung Memorial Hospital, Chiayi, Taiwan; ^9^ Department of Respiratory Care, Chang Gung University, Taoyuan, Taiwan; ^10^ Graduate Institute of Biomedical Sciences, Chang Gung University, Taoyuan, Taiwan; ^11^ Program for the Clinical Drug Discovery from Botanical Herbs, College of Pharmacy, Taipei Medical University, Taipei, Taiwan; ^12^ Chronic Diseases and Health Promotion Research Center, Chang Gung University of Science and Technology, Chia-Yi, Taiwan; ^13^ Research Center for Industry of Human Ecology and Research Center for Chinese Herbal Medicine, College of Human Ecology, Chang Gung University of Science and Technology, Taoyuan, Taiwan

**Keywords:** artocarpin, pro-oxidation, lung cancer, p53, apoptosis

## Abstract

Artocarpin has been shown to exhibit cytotoxic effects on different cancer cells, including non-small cell lung carcinoma (NSCLC, A549). However, the underlying mechanisms remain unclear. Here, we explore both p53-dependent and independent apoptosis pathways in artocarpin-treated NSCLC cells. Our results showed that artocarpin rapidly induced activation of cellular protein kinases including Erk1/2, p38 and Akt*^S473^*. Inhibition of these protein kinases prevented artocarpin-induced cell death. Moreover, artocarpin-induced phosphorylation of these protein kinases and apoptosis were mediated by induction of reactive oxygen species (ROS), as pretreatment with NAC (a ROS scavenger) and Apocynin (a Nox-2 inhibitor) blocked these events. Similarly, transient transfection of p47*^Phox^* or p91*^Phox^* siRNA attenuated artocarpin-induced NADPH oxidase activity and cell death. In addition, p53 dependent apoptotic proteins including PUMA, cytochrome c, Apaf-1 and caspase 3 were activated by artocarpin, and these effects can be abolished by antioxidants, MAPK inhibitors (U0126 and SB202190), but not by PI3K inhibitor (LY294002). Furthermore, we found that artocarpin-induced Akt phosphorylation led to increased NF-κB activity, which may act as an upstream regulator in the c-Myc and Noxa pathway. Therefore, we propose that enhancement of both ERK/ p38/ p53-dependent or independent Akt*^S473^*/NF-κB/c-Myc/Noxa cascade by Nox-derived ROS generation plays an important role in artocarpin-induced apoptosis in NSCLC cells.

## INTRODUCTION

Lung cancer, which causes about 1.59 million deaths a year, is a very common cancer and a major cause of death worldwide [1]. Apoptosis, a form of programmed cell death, plays animportant biological role in homeostasis. Insufficient apoptosis may result in uncontrolled cell proliferation and has been shown to be involved in various diseases, such as cancer [2–4]. Chemotherapy and γ-irradiation are major therapeutic modalities to decrease cancer cell proliferation in clinical medicine, but these therapies may also kill normal cells and lead to many side effects in human body, such as loss of immunity. Erridge et al. revealed that the five-year survival rate in lung cancer cases is less than 15% due to patient resistance to γ-radiation, chemotherapy and surgical intervention [5, 6]. Therefore, many researchers and pharmaceutical industries have been enthusiastically developing novel anticancer agents to increase the survival rate and improve life quality of lung cancer patients [7–9].

To the best of our knowledge, reactive oxygen species (ROS) plays a double-edged role in free radical biology and medicine [10–12]. On one hand, under normal physiologic conditions, the generation of ROS have important roles inphagocytosis, cell signaling and homeostasis, and ROS are subsequently eliminated by the scavenging system in normal cells [13]. On the other hand, under oxidative stress conditions, greater ROS amounts may oxidize the cellular lipids, proteins and DNA, leading to the aggravation of many clinical diseases, including inflammation, aging, cancer, neurodegenerative and cardiovascular diseases [14–16]. During the last decade, some anticancer drugs in clinical practice, such as platinum [17], paclitaxel [18], resveratrol [19], EGCG [20] and curcumin [21], have been shown to increase the level of NADPH oxidase (Nox)-derived ROS production to suppress cancer cell growth through mediating p53-independent apoptotic signaling components, including MAPKs (p38, ERK1/2 and JNK), PI3K/Akt, BAX/Bcl2 ratio, cytochrome c, Apaf-1, caspase cascade (caspase 3, 8 and 9) and PARP activation. Increasing evidence has also demonstrated that the mitogen activated protein kinase (MAPK) family is involved in p53-independent apoptosis [20–23]. In addition, Wartenber et al. indicated that overexpressing Nox-1 can increase ROS production and diminish the function of P-glycoprotein (an ABC cassette transporter which is involved in chemotherapeutic resistance), and finally overcome cancer drug resistance [24]. According to these viewpoints, a novel anticancer drug functioning as a Nox-derived ROS activator may have therapeutic effects in various types of cancer [25–27].

Artocarpus species are mainly grown in Southeast Asia and also extensively used in food, traditional medicine, agriculture and industry. Artocarpin, an isoprenyl flavonoid, is abundantly found in Artocarpus species, such as *Artocarpusaltilis* and *Artocarpusheterophylli*, andhas been reported to possess many pharmacological activities, including antityrosinase [28], antibacterial [28], 5α-reductase inhibitor [29], photoprotective [30], and anticancer properties [31]. In particular, Wang et al. [32] reported that artocarpin has a powerful cytotoxic effect on various human cancer cells, including non-small cell lung carcinoma (NSCLC, A549), breast adenocarcinoma (MCF-7), ovarian carcinoma (1A9), glioblastoma (U87-MG) and epidermoid carcinoma of the nasopharynx (KB). Until now, however, the molecular biological mechanisms by which artocarpin induce cancer cell apoptosis has not been clearly clarified.

This is the first study to evaluate the anticancer mechanisms of artocarpin on human non-small cell lung carcinoma cells. Here, we hypothesized that artocarpin induces apoptosis of non-small cell lung carcinoma cells via Nox-derived ROS generation. Furthermore, the ROS-mediated signaling contributes to activation of p38 MAPK, ERK1/2 or PI3K/Akt/NF-κB, which regulates p53-dependent or independent cell apoptosis.

## RESULTS

### Effects of artocarpin on cell proliferation and survival *in vitro*

**Figure 1:**
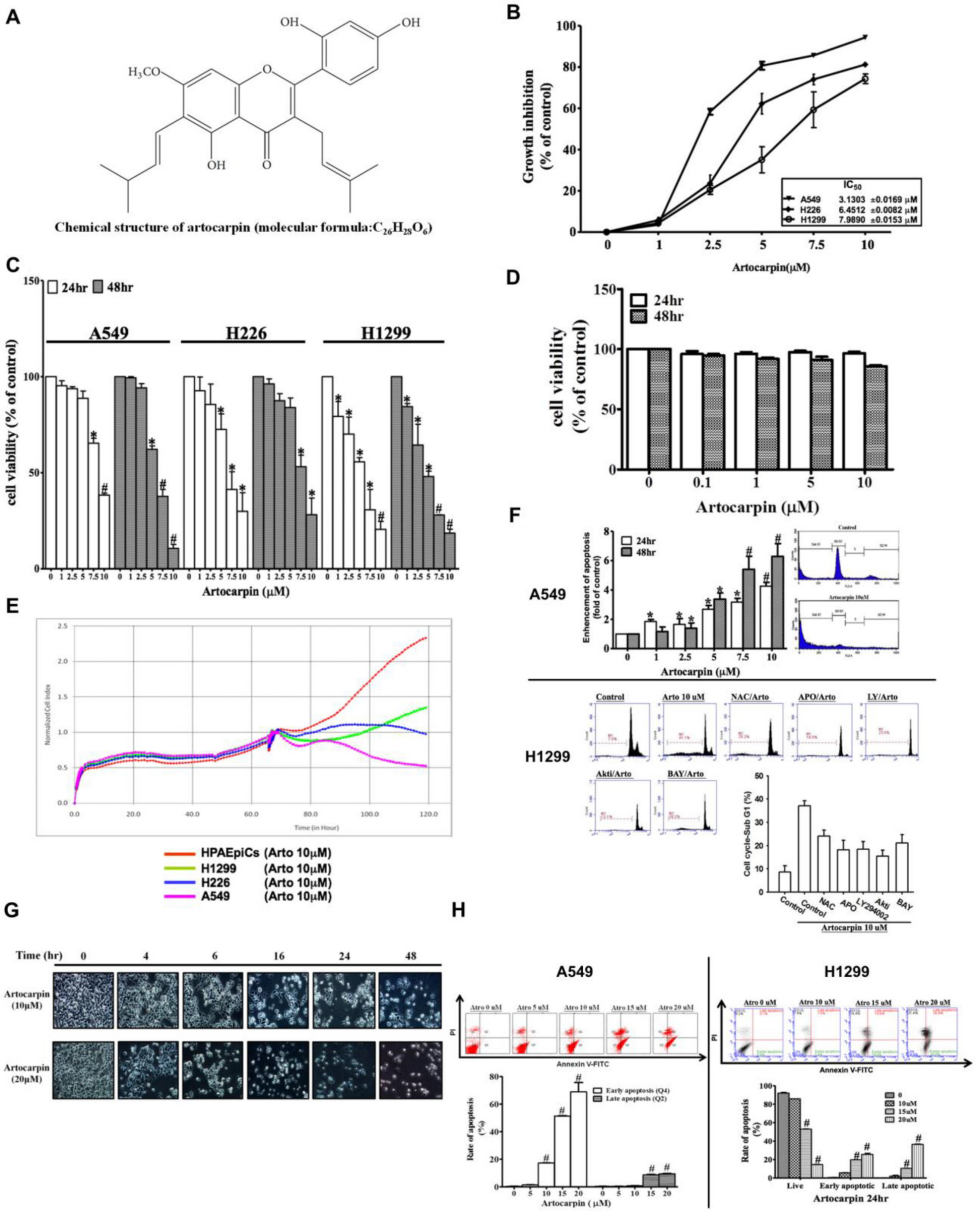
Growth inhibition of NSCLC cell lines by artocarpin (**A**) Chemical structure of artocarpin. (**B**) A549, H226 and H1299 cells were treated with different concentrations of artocarpin for 24 h. Inhibition of cell growth wasevaluated using the SRB assay. (**C**) A549, H226, H1299cells and (**D**) HPAEpiCs were treated with the indicated concentrations of artocarpin for 24 and 48 h. Cytotoxicity was evaluated using the MTT assay. Data shown are means ± SEM of at least three independent experiments. *P < 0.05, *^#P^* < 0.01 compared with the control group. (**E**) Real-time cytotoxicity assay to assess the time-dependent effect of artocarpin on cell viability in HPAEpiCs, H1299, H226 and A549 cells. Artocarpin was added at the 65 hour time point. (**F**) Following treatment with different concentrations of artocarpin for 24 h, apoptosis induction in A549 cells was evaluated by measuring the amounts of oligonucleosomal DNA fragmentation using the Cell Death ELISA*^PLUS^* kit. In addition, cell cycle analysis was performed in A549 and H1299 cells using flow cytometry. H1299 cells were also pretreated with the inhibitors NAC, APO, LY294002, Akti, and Bay117082. Data shown are means ± SEM. *P < 0.05, *^#P^* < 0.01, compared with the control group. (**G**) Morphological changes in A549 cells were observed by light microscopy. (**H**) After incubation with 0–20 μM artocarpin for 24 h, A549 and H1299 cells were stained with Annexin-V-FITC and PI for 15 min, and then evaluated by flow cytometry. Each bar represents the mean ± SD (*n* = 3). *P < 0.05, *^#P^* < 0.01 compared with the control group.

We first determined the anti-proliferative activities of artocarpin (Figure 1A) in the human NSCLC cell lines A549, H226 and H1299 using the sulforhodamine B (SRB) assay. Artocarpin inhibited cell proliferation in a concentration-dependent fashion, with IC_50_ values as shown in Figure 1B. Cytotoxicity was determined by the MTT assay after 24 or 48 h of treatment with artocarpin. When incubated with artocarpin (1–10 μM), the growth of A549, H226 and H1299 cells was markedly inhibited in a concentration-dependent manner (Figure 1C). To confirm the selectivity of the cytotoxic effect, nontumorous cells, such as human pulmonary epithelial cells (HPAEpiCs), were also evaluated. However, artocarpin showed much less activity against human pulmonary epithelial cells (Figure 1D and 1E). These data indicate that A549, H226 and H1299 cells were much more sensitive to the cytotoxic effects of artocarpin, in comparison with normal cells. In addition, cell death detection ELISA*^PLUS^* assay revealed that artocarpin induced DNA fragmentation in A549 cells. Moreover, increased proportion of cells in subG1 phase was observed in the artocarpin-treated cells (Figure 1F). In H1299 cells, the artocarpin-induced increase in subG1 phase cells was suppressed by pretreatment with the inhibitors NAC, APO, LY294002, Akti, and Bay117082. Cell morphology was captured by phase-contrast images after treatment with 10 and 20 μM of artocarpin for 24 h or 48 h. The morphological analysis revealed prominent cytotoxicity in artocarpin-treated A549 cells (Figure 1G). Moreover, the Annexin-V-FITC/PI assay showed induction of apoptosis following artocarpin exposure in A549 and H1299 cells. Representative results of Annexin-V-FITC/PI assay are presented in Figure 1H. Under control conditions, the majority of cells were viable cells (Annexin-V-negative/PI-negative). Following treatment with various concentrations of artocarpin for 24 h, the proportion of viable cells was decreased, while the proportionsof cells in early apoptosis (Annexin-V-positive/PI-negative) and late apoptosis (Annexin-V-positive/PI-positive) were increased. All tested concentrations of artocarpin could induce early apoptosis, while only 15 and 20 μM could significantly induce late apoptosis. The results demonstrated that artocarpin induced apoptosis of A549 and H1299 cells in a concentration-dependent manner, particularly early apoptosis (Figure 1).

### Artocarpin-induced apoptosis is associated with generation of ROS

**Figure 2:**
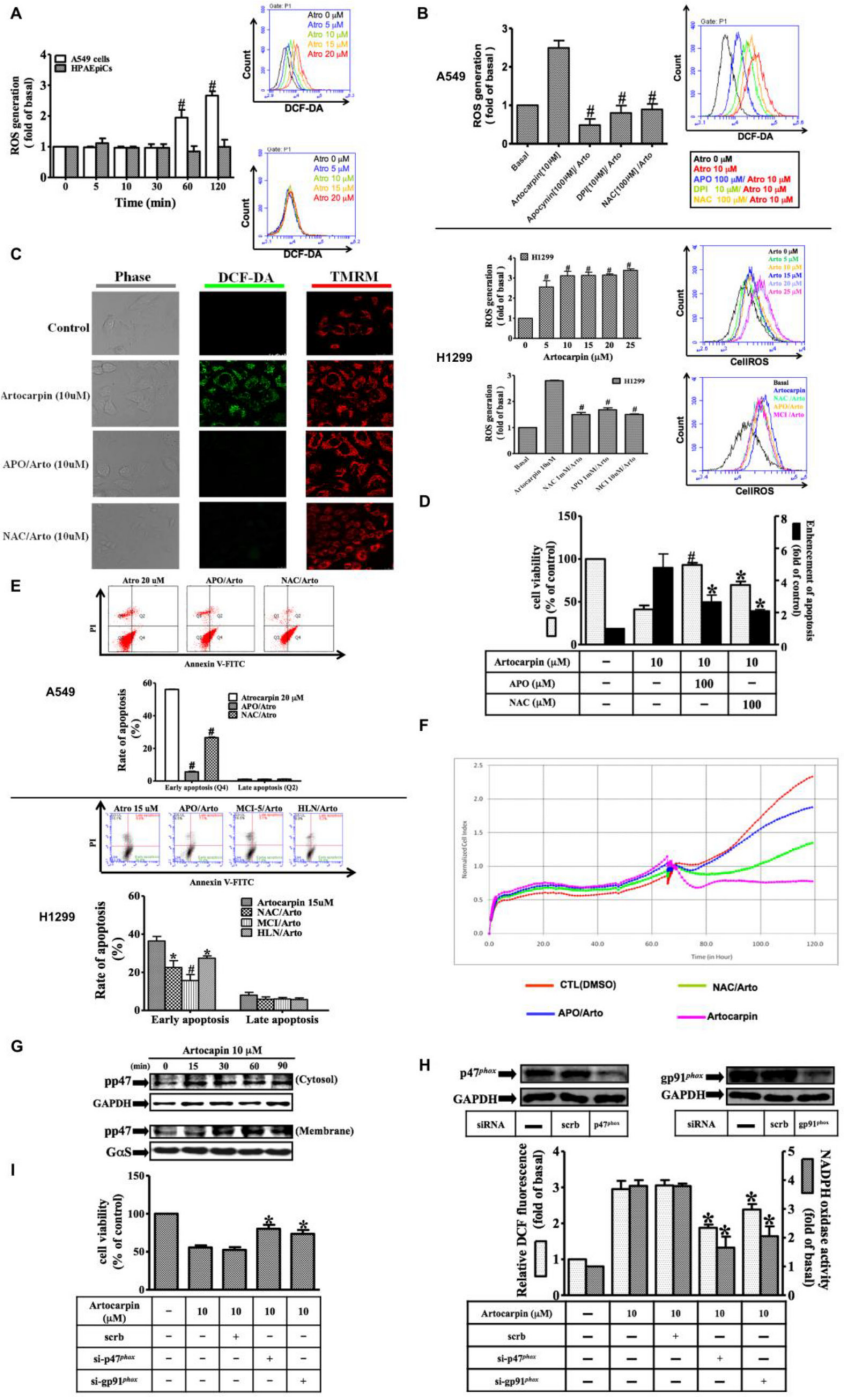
Artocarpin-induced activation of NADPH oxidase and generation of ROS triggers apoptosis in tumor cells Time dependence of artocarpin-induced ROS generation and p47*^phox^* activation are shown. (**A**) A549 cells and HPAEpiCs were stained with DCF-DA (10 μM) and treated with artocarpin for different periods of time. The fluorescence intensity was then determined. (**B**) A549 and H1299 cells were pretreated with DPI (10 μM), APO (100 μM), or NAC (100 μM) for 1 h and then stimulated with 10 μM artocarpin for 120 min. ROS generation was determined via fluorescent plate reader and flow cytometry. (**C**) Cells were stained with DCF-DA (10 μM) and TMRM (10 μM) and then pretreated with APO or NAC for 1 h before artocarpin administration. The ROS generation (green color) and mitochondrial membrane potential (red color) were detected via confocal microscope.(**D**–**F**)A549 cells were pretreated with APO or NAC for 1h, and then incubated with 10 μM artocarpin for 24h. Cytotoxicity was determined using MTT assay, flow cytometry(cells stained with Annexin-V and PI) and real-time cytotoxicity assay. (**G**) The membrane (ME) and cytosolic (CE) fractions were collected and subjected to Western blot analysis with an anti-phospho-p47*^phox^* antibody. (**H**)A549 cells were transfected with siRNAs for scrambled, Nox2 (gp91*^phox^*) or p47*^phox^*, and then treated with 10 μM artocarpin for 120 min. ROS generation (open bars) and Nox activity (shaded bars) were determined via ELISA reader. (**I**) A549 cells were transfected with siRNAs of Nox2 (gp91*^phox^*) or p47*^phox^*, and then treated with 10 μM artocarpin for 24 hr. Cytotoxicity was determined using MTT assay. (A–I) are means ± SEM. *P < 0.05, *^#P^* < 0.01, compared with the control group.

Accumulating studies have reported that various natural products exhibited powerful anti-tumor effects by generation of reactive oxygen species (ROS) with their pro-oxidative activities [25]. ROS are known to induce oxidative stress andDNA damage, and may act as a mediator of apoptosis. It is not known whether this form of pro-oxidative action of artocarpin occurs in A549 and H1299 cells. The intracellular levels of ROS induced by stimulation of A549 and H1299 cells with 10 μM artocarpin were measured using a fluorescent probe, dichlorofluorescin diacetate (DCF-DA). Cells were first stained with DCF-DA, incubated with artocarpin for the indicated times, and then the fluorescence emission intensity at 530 nm was determined following excitation at 485 nm. The fluorescence was evaluated via flow cytometer, ELISA reader or confocal microscope. In addition, the Nox activity in lung cancer cells was evaluated by lucigenin chemiluminescence and measured using a luminometer. As illustrated in Figure 2A, artocarpin induced ROS production in A549 and H1299 cells in a time and dose-dependent manner, however, the formation of ROS was not seen upon artocarpin stimulation of HPAEpiCs. Pretreatment with APO (a Nox2 inhibitor), DPI (a Nox inhibitor) or NAC (a ROS scavenger) significantly decreasedartocarpin-induced ROS generation in A549 and H1299 cells (Figure 2B), and similar findings were shown from the confocal microscope (Figure 2C). Image fluorescence from mitochondrial membrane potential dye (TMRM, Figure 2C) showed that mitochondrial membrane potential was not changed after 2h of artocarpin exposure, suggesting that ROS generation may not be directly related to the mitochondria at this time point. We further found that in A549 and H1299 cells, treatment with artocarpin resulted in increased mitochondrial superoxide level at the 24 hour time point, which was partially suppressed by pretreatment with MitoTEMPO (a specific scavenger for mitochondrial superoxide anions) (Supplementary Figure 1). In addition, artocarpin-induced apoptosis in A549 and H1299 cells can be attenuated by pre-treatment with MitoTEMPO (Supplementary Figure 2). Moreover, pretreatment with APO or NAC markedly inhibited artocarpin-induced apoptosis as observed with the MTT, DNA fragmentation, real-time cytotoxicity and Annexin-V-FITC/PI assays (Figure 2D–2F). These data indicated that Nox2-mediated ROS generation may participate in artocarpin-induced oxidative damage to DNA and apoptosis in A549 and H1299 cells. Furthermore, previous studies have shown that during the process of Nox2 activation, the p47*^phox^*cytosolic subunit is phosphorylated and translocates with the p67*^phox^*subunit to the cell membrane, and combine with the gp91*^phox^* (Nox2) subunit to form an active enzyme complex [33]. Therefore, we found that artocarpin induced phosphorylation of p47*^phox^* in a time dependent manner (Figure 2G), and promoted p47*^phox^* translocation from the cytoplasm to the cell membrane in 30 min. Additionally, transfection with siRNAs for Nox2 (gp91*^phox^*) and p47*^phox^* effectively attenuated artocarpin-induced ROS formation and Nox activity (Figure 2H), which in turn inhibited apoptosis of A549 cells (Figure 2I). These results indicated that artocarpin-induced DNA damage and cell apoptosis are mediated via Nox2/ p47*^phox^* dependent ROS production in A549 cells.

### Involvement of ROS-dependent MAP kinases and PI3K/Akt pathways in apoptosis induction in A549 and H1299 cells by artocarpin

**Figure 3:**
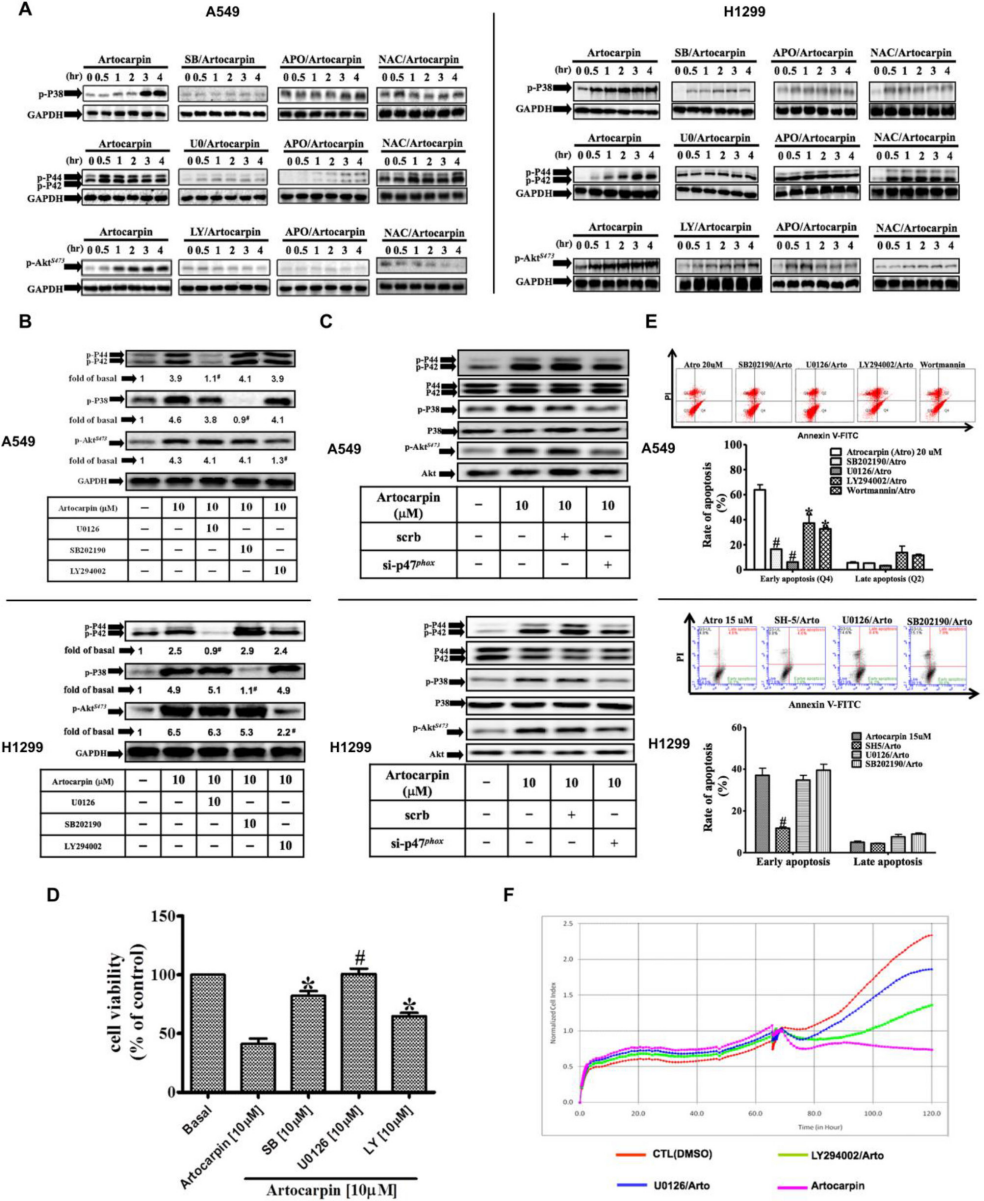
ROS-dependent MAPKs activation is involved in artocarpin-induced apoptosis in A549 and H1299 cells Cells were treated with 10 μM artocarpin for different periods of time, followed by Western blot analysis using antibodies against phospho-p38 MAPK, phospho-ERK1/2 or phospho-Akt. (**A**) Western blot analysis showing the effects of 10 μM SB202190 (p38 inhibitor), 10 μM U0126 (ERK1/2 inhibitor), 10 μM LY294002 (PI3K inhibitor), APO (100 μM) or NAC (100 μM) on the phosphorylation of p38 MAPK, ERK1/2 or Akt in artocarpin-treated A549 and H1299 cells. The blots were probed for GAPDH as a loading control. (**B**) Cross talk between MAPKs and Akt phosphorylation in artocarpin-treated A549 and H1299 cells. Cells were pretreated with 10 μM SB202190, 10 μM U0126 or 10 μM LY294002 for 1hr and then treated with 10 μM artocarpin for 4 hr. (**C**) Relationship between MAPKs, Akt and p47*^phox^* in artocarpin-treated A549 and H1299 cells. Cells were transfected with either scrambled siRNA or p47*^phox^* siRNA and then treated with 10 μM artocarpin for 4 hr. (**D**, **E**) Cells were pretreated with10 μM SB202190, 10 μM U0126 or 10 μM LY294002 for 1 h, and then treated with 10 μM artocarpin for 24h. Cytotoxicity was determined using MTT assay and stained with Annexin-V and PI as previously described in Figure 1. Data shown are means ± SEM. *P < 0.05, *^#P^* < 0.01, compared to artocarpin treatment alone. (**F**) Real-time cytotoxicity assay to assess the effect of U0126 and LY294002 on artocarpin-induced cell cytotoxicity in A549 cells. Artocarpin was added at the 65 hour time point.

Previous studies have shown that ROS formation can elicit changes in the phosphorylation of MAPKs or Akt [21, 34], and that MAPKs or Akt regulate apoptosis [21, 22, 34, 35]. Therefore, we first examined whether MAPKs and Akt are also involved in artocarpin-induced apoptosis in A549 and H1299 cells. The effects of artocarpin on the phosphorylation of MAPKs and Akt*^S473^* were evaluated by Western blot analysis. As shown in Figure 3A, artocarpin induced phosphorylation of p38 MAPK, ERK1/2 and Akt*^S473^*, but not JNK1/2 (data not shown) in a time dependent manner, and these effects were significantly attenuated by their specific inhibitors as well as APO and NAC. We also used specific inhibitors to further evaluate the role of these pathways and the inter-relationship between them. As shown in Figure 3B, phosphorylation of p38 MAPK, ERK1/2 and Akt*^S473^* stimulated by artocarpin for 4 hours was suppressed by pretreatment with the specific inhibitors U0126, SB202190 and LY294002, respectively. Moreover, inhibition of one signaling kinase did not affect the other kinases, indicating that phosphorylation of p38 MAPK, ERK1/2 and Akt*^S473^* by artocarpin occurred in parallel. As shown in Figure 3C, artocarpin-induced phosphorylation of ERK, p38 and Akt was significantly suppressed by transfection with p47*^phox^* siRNA but not scrambled siRNA. This indicates that artocarpin-induced phosphorylation of ERK, p38 and Akt was mediated through the p47*^phox^* pathway. Additionally, pretreatment with U0126 and SB202190 for 1 hour markedly inhibited artocarpin-induced cytotoxicity (Figure 3D), early apoptosis (Figure 3E) and real-time cytotoxicity (Figure 3F), and such effects were also partially significantly reduced by pretreatment with LY294002 and Wortmannin (inhibitors of PI3K). These results suggest that ROS plays a critical role in artocarpin-induced p38 MAPK, ERK1/2 and Akt*^S473^* phosphorylation, leading to the induction of cytotoxicity and apoptosis in A549 and H1299 cells.

### ROS mediated MAPKs activation contributes to artocarpin-elicited p53 activation, PUMA and Cytochrome C expression, and apoptosis induction

**Figure 4:**
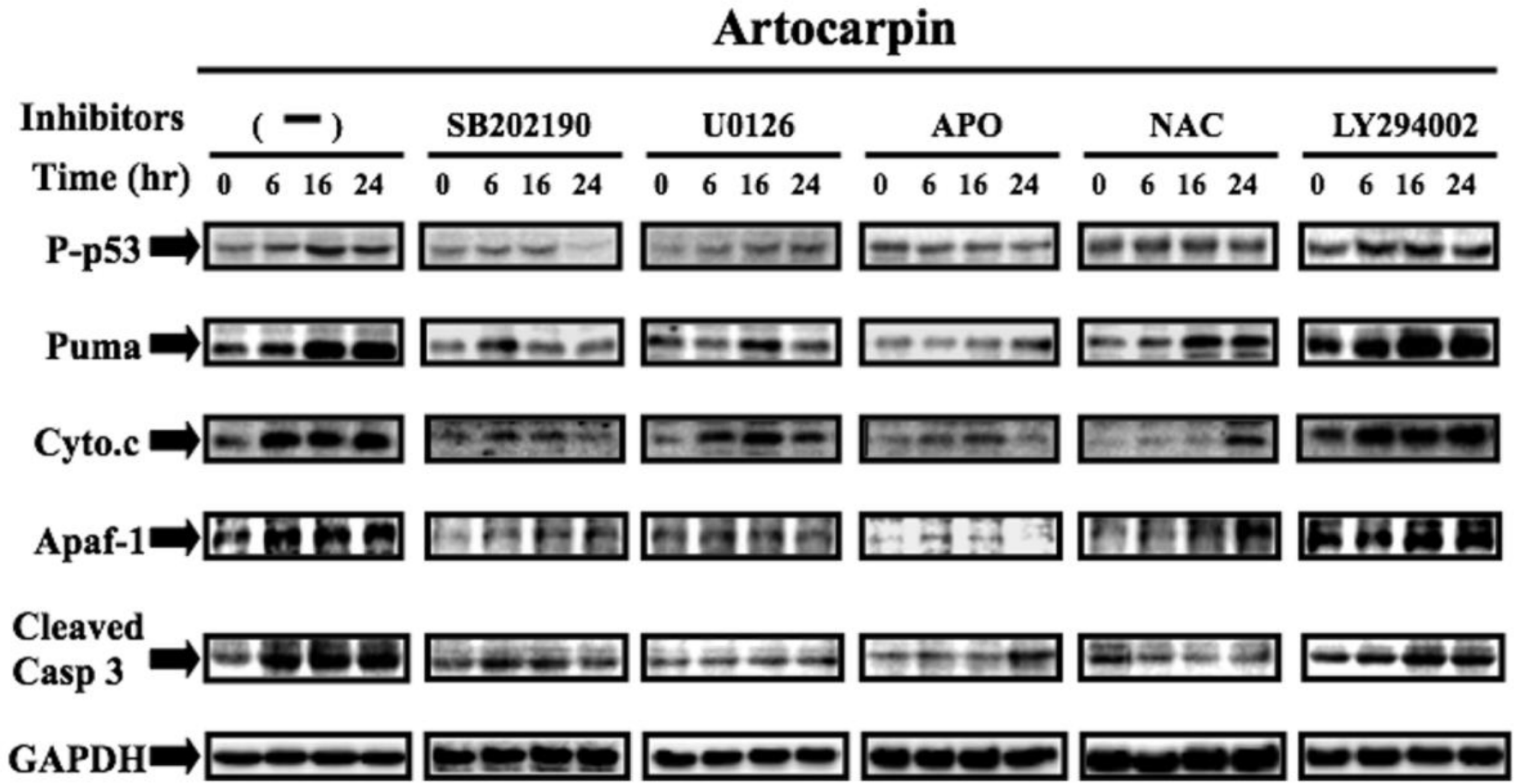
Artocarpin up-regulated the expression of p53-dependent apoptotic proteins via p38 MAPK and ERK1/2 pathway, but not by Akt pathway A549 cells were treated with 10 μM artocarpin for various periods of time, followed by Western blot analysis using antibodies against phospho-p53, PUMA, Cytochrome C, Apaf-1and caspase-3. Western blot analysis also demonstrating the effects of 10 μM SB202190, 10 μM U0126, 10 μM LY294002, APO (100 μM) or NAC (100 μM) treatment on the up-regulation of phospho-p53, PUMA, Cytochrome C, Apaf-1and caspase-3 in artocarpin-treated A549 cells. The blots were probed for GAPDH as a loading control. Each blot is representative of three independent experiments, all of which had similar results.

MAPKs have been shown to activate p53 in response to different stressful stimuli, and the phosphorylation of p53 may lead to cell cycle arrest and apoptosis [36, 37]. To examine whether MAPKs activation plays a role in artocarpin-induced p53 phosphorylation and downstream apoptotic events, A549 cells were pretreated with antioxidants, MAPK inhibitors (U0126, SB202190) and PI3K inhibitor (LY294002) for 1 h prior to artocarpin incubation. The levels of p53, PUMA, Cytochrome C, Apaf-1 and cleaved-caspase 3 were evaluated by Western blotting. As shown in Figure 4, artocarpin promoted phosphorylation of p53 and expression of PUMA, Cytochrome C, Apaf-1 and cleaved-caspase 3 in a time dependent manner, and these effects can be decreased by pretreatment with antioxidants, SB202190 and U0126, but not by LY294002. We also performed experiments to show that treatment of A549 cells with artocarpin resulted in increased caspase-3, caspase-7 and caspase-9 activity, which were attenuated by pre-treatment with APO, NAC, MCI, U0126 and SB202190 (Supplementary Figure 3). These data indicate that artocarpin induction of ROS leads to activation of p38 and ERK1/2 pathways and partial activation of PI3K/ Akt*^S473^*, thereby leading to p53-dependent or independent apoptosis of tumor cells.

### PI3K/Akt-dependent activation of NF-kB and expression of c-Myc/Noxa are required for artocarpin-induced apoptosis of A549 and H1299 cells

**Figure 5:**
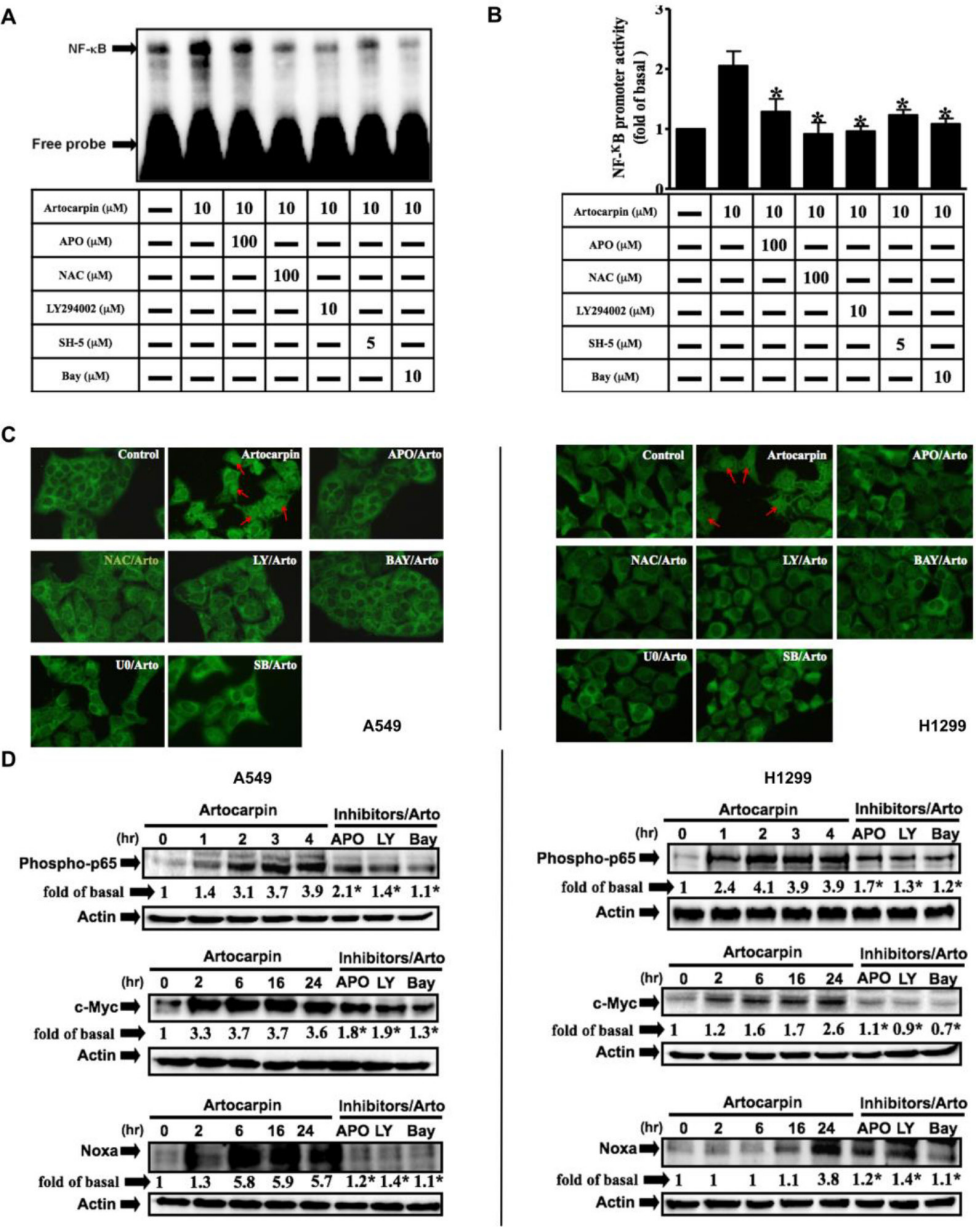
Artocarpin induced activation of NF-kB and expression of c-Myc/Noxa via Akt pathway (**A**) Nuclear extracts from untreated cells or cells pretreated with APO, LY294002 or BAY117082 for 1hr followed by incubation with 10 μM artocarpin for 4 h were tested for NF-kB DNA binding activity by EMSA. (**B**) A549 cells were pretreated with APO, LY294002 or BAY117082 for 1hr, then treated with 10 μM artocarpin for 4 h were tested for NF-kB transcription activity by reporter gene assay. *P < 0.05 compared to artocarpin treatment alone. (**C**) Fluorescence immunocytochemistry to assess P65 nuclear translocation. A549 and H1299 cells were pretreated with APO, NAC, LY294002, BAY117082, U0126 or SB202190 for 1 h, and then treated with 10 μM artocarpin. (**D**) A549 and H1299cells were pretreated with APO, LY294002 or BAY117082 for 1 h, and then treated with 10 μM artocarpin for the indicated times. The nuclear extract levels of phospho-p65, c-Myc and Noxa proteins were evaluated by Western blotting. (A–D) Each blot is representative of three independent experiments, all of which had similar results.

To elucidate the pathways involved in regulation of artocarpin-induced apoptosis by PI3K/Akt pathway, we evaluated the activation status of NF-κB, which regulates the transcription of DNA and is delicately susceptible to cellular oxidative stress [38]. NF-κB activation by artocarpin was investigated by Gel-shift assay, promoter assay and P65 nuclear translocation assay(fluorescence immunocytochemistry). As shown in Figure 5A–5C, NF-kB binding activity, promoter activity and P65 nuclear translocation were low in untreated controls but weresignificantly increased following treatment with artocarpin in A549 cells. Pretreatment with APO, NAC, LY294002, SH-5, Bay117082, U0126 and SB202190 blocked the artocarpin-stimulated increase in NF-kB activity. Furthermore, the nuclear fraction was prepared to determine whether artocarpin increased nuclear translocation of the NF-kB p65 subunit. As shown in Figure 5D, artocarpin stimulated rapid phosphorylation and translocation of p65 in A549 and H1299 cells, which was significantly suppressed by pretreatment with APO, LY294002 and Bay117082. Finally, we confirmed that accumulation of c-Myc (a transcription target of NF-kB) and Noxa (a transcription target of c-Myc) proteins was due to NF-kB activation. As expected, artocarpin induced expression of c-Myc and Noxa in a time dependent manner, and these effects were significantly suppressed by APO, LY294002 and Bay117082 (Figure 5D). These results demonstrated that NF-kB/c-Myc/Noxa was involved in cell apoptosis mediated through Nox/ROS generation following the activation of PI3K/Akt in A549 and H1299 cells.

### Artocarpin suppressed lung cancer growth in the mouse xenograft model

**Figure 6:**
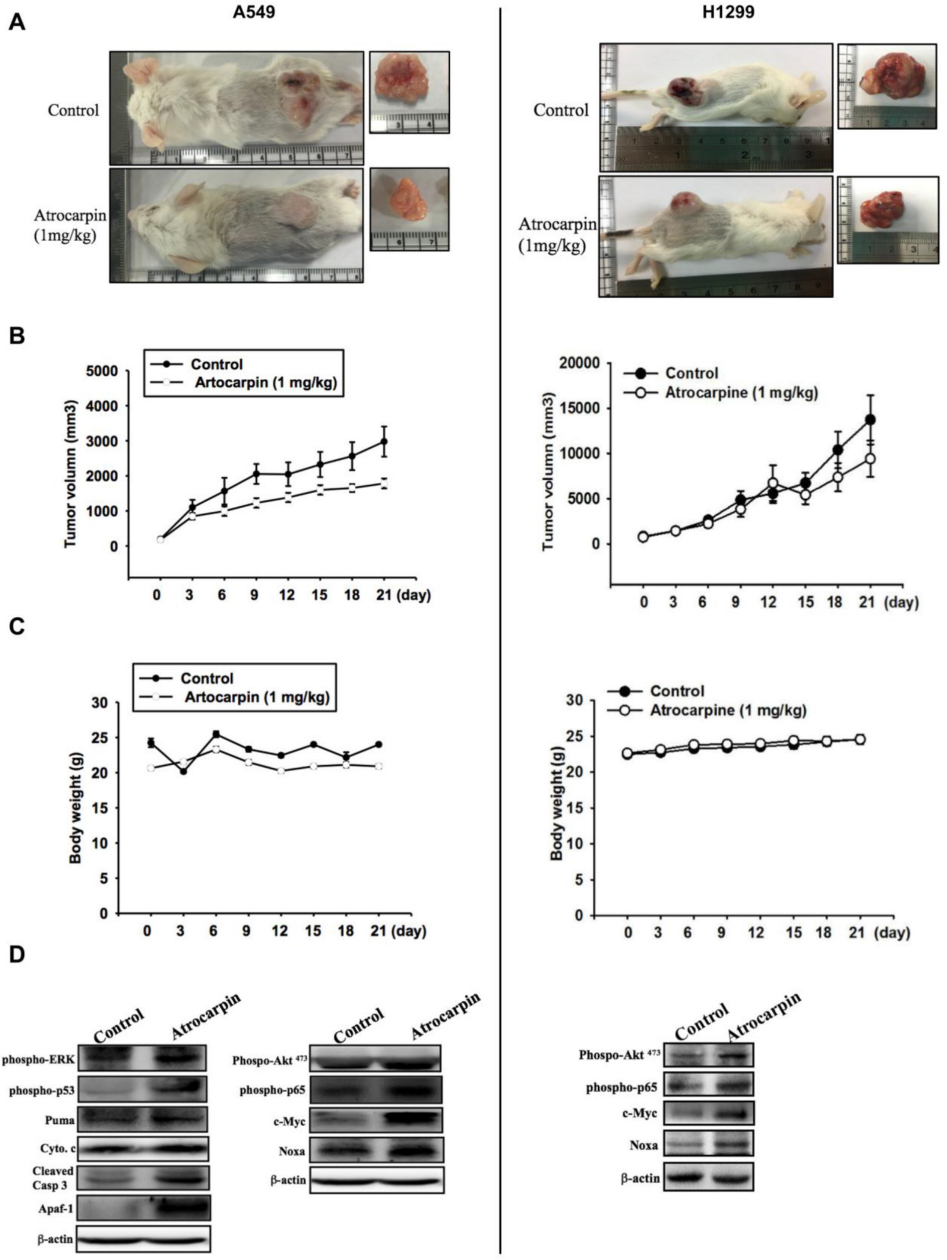
Artocarpin inhibits xenograft tumor growth in SCID mice (**A**, **B**) Mice were subcutaneously injected with A549 or H1299 cells. When the tumors grew to 100 mm*^3^* in size, the mice were administered artocarpin (1 mg/kg) or vehicle once a day for 3 weeks. Tumor volume was determined at various times following tumor implantation (*n* = 8–10). (**C**) Mice body weight was evaluated at various times after tumor implantation. (**D**) Western blot analysis for levels of ERK, p53, PUMA, Cytochrome C, Apaf-1, caspase 3, Akt*^S473^*, p65, c-Myc and Noxa in tumors with and without artocarpin treatment.

To evaluate whether artocarpin shows anticancer properties *in vivo*, we implanted xenografts of A549 and H1299 cells into SCID mice. When the xenograft tumors grew to 100 mm*^3^* in size, the mice were allocated into two groups, the first group treated withvehicle and the second group treated with artocarpin (1 mg/kg/day). Artocarpin significantly inhibited tumor growth (Figure 6B). The mean tumor volume in artocarpin-treated mice was significantly reduced compared with vehicle-treated control mice(Figure 6A). Western blot analysis of excised tumor tissues *ex vivo* revealed significant increases in ERK, p53, PUMA, Cytochrome C, Apaf-1, caspase 3, Akt*^S473^*, p65, c-Myc and Noxa expression in the artocarpin treated group compared with tumors from the control group (Figure 6D). These findings indicate that artocarpin suppressed tumor growth by promoting apoptosis of A549 and H1299 cells *in vivo*.

## DISCUSSION

In this study, we clearly demonstrate that artocarpin dose-dependently suppressed the proliferation of non-small cell lung cancer (NSCLC) cell lines, but showed much less activity in normal human pulmonary epithelial cells (HPAEpiCs). We also found that artocarpin induced formation of ROS and promoted apoptosis only in NSCLC cell lines, but showed no significant effects in normal cells. These results are consistent with findings in other bioactive compounds and plant secondary metabolites, such as curcumin [21], EGCG [39] and resveratrol [19]. To our knowledge, this is the first study to show that artocarpin induces ROS generation and consequently causes DNA oxidative damage in tumor cells.

Artocarpus species (Moraceae) are evergreen trees distributed over tropical regions of Asia and are extensively used in food, traditional folk medicines, agriculture and industry. Artocarpin is a prenylated flavonoid derived from Artocarpus species. The chemical structure of artocarpin contains lipophilic isoprenoid groups, which increases its affinity to biological membranes [40]. Previously, artocarpin has been shown to exhibit cytotoxic activity against cancer cells. The results of the present study revealed that the IC_50_ of artocarpin in three NSCLC cell lines at 24 h was 3 to 8 μM, which was much lower than that of many natural anticancer compounds, such as curcumin (about 78 μM), resveratrol (about 33 μM) and EGCG (about 68 μM) [41–43]. How artocarpin exerts its powerful anticancer activities on A549 and H1299 cells have not been reported. It is important to elucidate the mechanisms involved in artocarpin-induced cell death.

Accumulating investigations have demonstrated that ROS acts as a mediator of apoptosis. ROS species, including free radicals such as superoxide and hydroxyl radicals, are produced by multiple cellular sources, such as the mitochondria and NADPH oxidases, lipoxygenases and cycloxygenases. In our study, we have established that after artocarpin treatment of A549 and H1299 cells, both Nox activity (Figure 2G) and ROS generation (Figure 2A–C and 2G) were significantly increased, and these effects were suppressed by pretreatment with inhibitors including APO (a Nox2 inhibitor) and NAC (a ROS scavenger) or by transfection with siRNAs for Nox2 (gp91*^phox^*) and p47*^phox^*. In fact, our data also provide evidence that tetramethyl rhodamine methyl ester (TMRM) is suitable for determining mitochondrial membrane permeability transition, which was not changed in response to artocarpin at the two hour time point (Figure 2B). These data indicated that the major source of ROS is NADPH oxidases and not the mitochondria. However, excessive amounts of ROS can induce oxidative damage to DNA, proteins, and lipids leading to cell death. We found that pre-incubation with antioxidants or siRNAs for Nox2 (gp91*^phox^*) and p47*^phox^*significantly attenuated artocarpin-induced DNA oxidative damage and cell apoptosis, as determined by MTT assay, Annexin-V-FITC/PI staining, DNA fragmentation and real-time cytotoxicity assay. Therefore, the findings of the present study indicate that artocarpin exerted powerful anti-tumor effects by targeting NADPH oxidases and generation of ROS with their pro-oxidative activities.

Previous studies have revealed that MAPKs or PI3K/Akt regulate cell growth, apoptosis, and inflammation, and may be activated following oxidative stress [25, 44]. Thus, we further investigated whether artocarpin-induced apoptosis is mediated by activation of MAPKs or PI3K/Akt pathways. In this study, we showed that artocarpin-induced phosphorylation of ERK1/2, p38 MAPK and Akt*473* are due to the induction of Nox2-derived ROS which were confirmed using APO and NAC. Next, we established that phosphorylation of ERK1/2, p38 MAPK and Akt*473* by artocarpin occurred in parallel. These findings are consistent with previous studies showing that distinct members of the MAPK family are activated in a ROS-dependent manner [45]. Interestingly, most studies have shown that Akt serves as a proliferation and anti-apoptotic signal. In our study, pretreatment of cellswith specific inhibitors of ERK1/2, p38 and Akt*473* significantly suppressed the growth of A549 and H1299 cells, indicating that ERK1/2, p38 and Akt*473* are required for artocarpin-induced apoptosis. Phosphorylated Akt at S473 is the main active form Akt, and similar findings were also reported by Wang et al. [46].

The tumor suppressor gene p53 often shows somatic mutations or deletions in various human NSCLC cells. Previous studies have shown that two of the most important signaling pathwaysinvolved in cancer are the MAPKs and PI3K/Akt pathways, which may contribute to both p53-dependent and p53-independent apoptosis [21, 46–49]. In this study, we noticed that artocarpin is able to induce apoptosis in not only p53-wild type tumor cells (A549), but also p53-mutant tumor cells (H1299 and H226) (Figure 1A and 1B).Moreover, we found that activation of p53 by artocarpin subsequently regulated downstream apoptotic proteins, including PUMA, Cytochrome C, Apaf-1 and caspase 3, and such effects were ameliorated by reduction of ROS and inhibition of ERK1/2 or p38 MAPK. However, blocking of PI3K/Akt pathway by LY294002 showed less effect on p53-dependent apoptotic pathway. Thus we consider that, although ERK1/2 or p38 MAPK play a major role in artocarpin-induced p53-dependent apoptosis, artocarpin may also induce PI3K/Akt signaling via a p53-independent pathway.

The transcription factor NF-kB is a major PI3K/Akt downstream effector, and plays a dual role as an attenuator or promoter of apoptosis. It regulates the transcription of DNA, and mediates apoptosis in response to oxidative stress [38]. Recent studies have demonstrated that ROS-dependent NF-kB activation induced the protein expression of c-Myc and Noxa in p53-independent human NSCLC cell death [50]. On the contrary, NF-kB activation was involved in resistance to oxidative stress and p53-mediated programmed cell death [51–53]. Therefore, we investigated whether this transcription factor may play a role in artocarpin-induced apoptosis. In the current study, we observed that artocarpin induced the activation of NF-kB via a Nox2/ROS/PI3K/Akt dependent signaling pathway. Correspondingly, amelioration of Nox2/ROS/PI3K/Akt pathway significantly attenuated artocarpin-induced translocation ofNF-kB and up-regulation of c-Myc and Noxa proteins. These results demonstrated that activation of the p53-independent ROS/NF-kB/c-Myc/Noxa signaling pathway by artocarpin plays a critical role in inducing apoptosis in A549 and H1299 cells.

**Figure 7:**
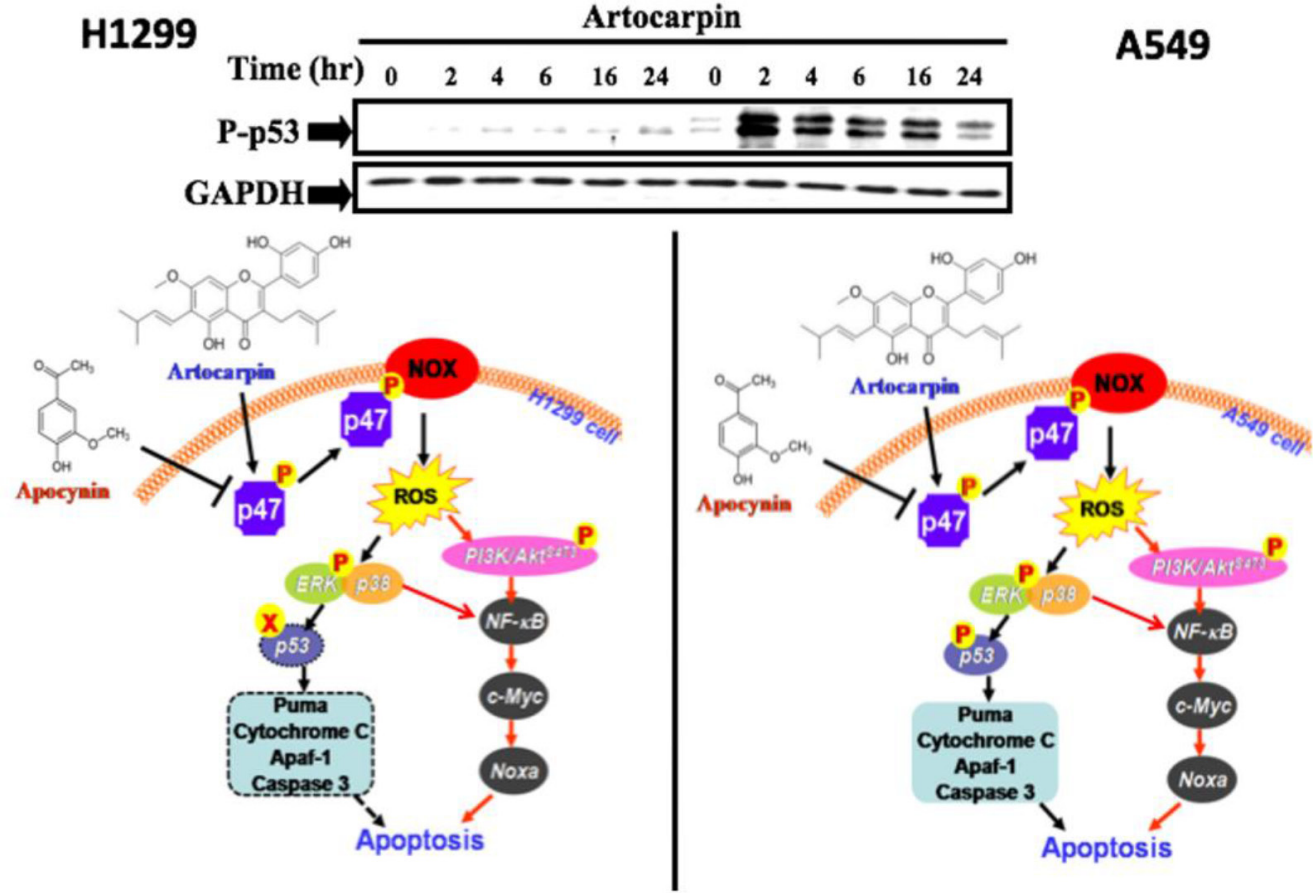
Schematic diagram showing the proposed signaling pathways involved in artocarpin-induced apoptosis in A549 and H1299 cells Artocarpin-induced apoptosis is mediated by activation of the Nox2/p47*^phox^*pathway leading to enhanced ROS production, which then induces the activation of two distinct signaling cascades, including ERK MAPK/ p38 MAPK/p53-dependent activation of PUMA/Cytochrome C/ Apaf-1/ caspase 3 pathway in A549 cells and PI3K/ Akt*^s473^*/ p53-independent activation of NF-kB/ c-Myc/Noxa pathway in both A549 and H1299 cells.

In conclusion, our results demonstrated that whereas artocarpin induces cytotoxic effects in human NSCLC cells, it may exert protective effects in normal HPAEpiCs. In addition, artocarpin may serve as a pro-oxidant only in human NSCLC cells, but not in normal HPAEpiCs. Previously, various flavonoids (including flavone acetic acid, quercetin, and flavopiridol) have entered human clinical trials, and have shown promising anticancer effects clinically [54]. We propose that cell apoptosis caused by artocarpin-induced oxidative stress and ROS generation can be an important mechanism for cancer prevention and therapy. Additionally, this study is the first to demonstrate that artocarpin-induced apoptosis is mediated through activation of the Nox2/p47*^phox^* pathway leading to enhanced ROS production, which then induces the activation of two distinct signaling cascades, including ERK MAPK/ p38 MAPK/p53-dependent activation of PUMA/Cytochrome C/ Apaf-1/ caspase 3 pathway in A549 cells and PI3K/ Akt*^s473^*/ p53-independent activation of NF-kB/ c-Myc/Noxa pathway in both A549 and H1299 cells, as shown in the composite scheme in Figure 7.

## MATERIALS AND METHODS

### Reagents and chemicals

DMEM/F-12 medium, FBS, and Lipofectamine 2000 were obtained from Invitrogen (Carlsbad, CA, USA). Western blotting materials (Hybond C membrane, enhanced chemiluminescence (ECL) system and Hyperfilms) were purchased from GE Healthcare Biosciences (Buckinghamshire, UK). The monoclonal antibodies cytochrome C, Apaf-1, caspase 3 and anti-phospho p65 were obtained from Santa Cruz Biotechnology (Santa Cruz, CA, USA). PhosphoPlus p53, ERK1/2, p38 and Akt antibodies were obtained from Cell Signaling Technology (Danvers, MA, USA), and phosphoPlus p47*^phox^* antibody was purchased from Assay Biotechnology (Sunnyvale, CA, USA). GAPDH antibody was obtained from Biogenesis (Boumemouth, UK). c-Myc and Noxa were purchased from Calbiochem, San Diego, CA, USA. N-acetylcysteine (NAC), apocynin (APO), Bay117082, U0126, SB202190, SH-5, LY294002 and wortmannin were from Biomol (Plymouth Meeting, PA, USA). Artocarpin (purity > 98% by high performance liquid chromatography analysis) was obtained from Pulin Biotech Company Limited (Taipei, Taiwan).

### Cell cultures

A549 cells(p53 wild-type) were obtained from the Food Industry Research and Development Institute (Taiwan) in the year 2012. p53 null H1299 and p53 mutant H226 cells were generously provided by Professor Mei-Chuan Chen (Program for the Clinical Drug Discovery from Botanical Herbs, College of Pharmacy, Taipei Medical University, Taiwan). HPAEpiCs were purchased from Cell Applications, Inc (San Diego, CA) in the year 2012. All cells were cultured in DMEM/F-12 with 10% fetal bovine serum (FBS) and antibiotics (100 U/ml penicillin G, 100 mg/ml streptomycin, and 250 ng/ml fungizone), and kept in an incubator with 5% CO2 at 37°C. For evaluation of protein expression and cell cytotoxicity, cells were seeded onto 12-well culture plates and 96-cm culture dishes, respectively. Cells from passages 5 to 20 were used in this study.

### Cell proliferation assay

Cell proliferation was evaluated using the sulforhodamine B (SRB) assay as described previously [55]. Following treatment with artocarpin with or without various inhibitors, viable cells were fixed with 10% trichloroacetic acid (TCA) and stained with 0.4% sulforhodamine B (SRB). After washing with 1% acetic acid, the cell-bound dye was dissolved with 10 mMTris-base. The absorbance was then measured by a microplate reader at 515 nm.

### Cell viability assay

The MTT assay was used for evaluation of cell viability. Lung cancer cells were seeded onto 96-well plates overnight. After treatment with different concentrations of artocarpin for 24 h or 48 h, MTT solution was added. Following incubation for one hour at 37°C, the plates were read using a microplate spectrophotometer at a wavelength of 550 nm.

### Cell apoptosis assay

The Cell Death Detection ELISA*^PLUS^* kit (Roche Diagnostics, Basel, Switzerland) was employed to evaluate artocarpin-induced apoptosis in accordance with the manufacturer's protocols. After artocarpin treatment for 24 h or 48 h, the amount of apoptotic oligonucleosomal DNA fragments was detected using an ELISA reader (absorption wavelength 405 nm, reference wavelength 490 nm).

### Real-time cytotoxicity assay

To determine the time-dependent cytotoxic effect of artocarpin on lung cancer cells, the xCELLigence Real-Time Cell Analyzer (ACEA Biosciences, San Diego, CA, USA) was also used [56]. Cell viability was measured over a 48 hour period following treatment with 10 μM artocarpin with or without inhibitors.

### Flow cytometric analysis

Evaluation of apoptosis was performed using an Annexin-V-FITC/propidum iodide (PI) apoptosis kit as described previously [51]. Following treatment with artocarpin with or without inhibitors, cells were labeled with Annexin-V and PI, and then evaluated by a flow cytometer.

### Cell transfection of siRNAs

The human p47*^phox^* siRNA (SC-29422), Nox2 siRNA (gp91*^phox^*, SC35503) and scrambled siRNA (SC-37007) were purchased from Santa Cruz Biotechnology (CA, USA). Transient transfections with various siRNAs (100 nM) wer eperformed using Lipofectamine transfection reagent in accordance with the manufacturer's protocols. The siRNA used in this study were labeled with EGFP. The transfection efficiency was evaluated by transfection with EGFP, and was determined to be approximately 60%.

### Western blotting

Cells were seeded onto 12-well plates and serum starved for 24 h. Following treatment with artocarpin (10 μM) with or without inhibitors or siRNAs for different periods of time, cells were collected and lysed in lysis buffer. The protein concentration was measured by the BCA kit. Proteins were separated using sodium dodecyl sulfate polyacrylamide gel electrophoresis (SDS-PAGE). Following transfer onto nitrocellulose membranes, the proteins were incubated with various antibodies overnight at 4°C, including phospho-p65, cytochrome c, p-ERK1/2, p-p38, p-AKT, caspase-3, phospho-p53 (Ser15), PUMA, Apaf-1, c-Myc and Noxa. Secondary anti-goat or anti-mouse horseradish peroxidase antibodies (1:2000 dilution) were then added for 1 h, and the bands were visualized using ECL reagents and developed by Hyperfilm-ECL.

### Nuclear protein extraction

Extraction of nuclear proteins was performed as described previously [55]. After artocarpin treatment, cells were collected, centrifuged at 400 × *g* for 5 min at 4°C. The supernatant representing the cytoplasmic protein fraction was discarded, and the pellet was centrifuged at 11000 × *g* for 10 min at 4°C. The final supernatant representing the nuclear protein fraction was stored at −20°C. Western blotting with p65, Noxa or c-Myc monoclonal antibodies were performed on the nuclear fractions.

### Isolation of cell fraction

The cytosolic and plasma membrane fractions were isolated as previously described [33]. Cells were lysed in lysis buffer, and then centrifuged at 16,000 g for 20 min at 4°C. The supernatant which represented the cytosolic fraction was collected. Following centrifugation, the pellet (representing the plasma membrane fraction) was collected. Western blotting with p47*^phox^* monoclonal mouse antibody was carried out on the plasma membrane fractions.

### Measurement of NF-kB luciferase activity

The NF-kB promoter activity was evaluated using the luciferase assay kit (Promega, Madison, WI, USA) as described previously [57]. Cells were transfected with the pNF-κB-Luc plasmid (Clontech). Following treatment, luciferase assay solution was added, and the luminescence was determined using a luminometer. The promoter luciferase activities were standardized to β -galactosidase.

### Measurement of NADPH oxidase activity

The NADPH oxidase (Nox) activity inlung cancer cells was evaluated by the lucigenin chemiluminescence assay as described previously [58, 59]. Following treatment, the cell membrane fraction was collected, NADPH (Sigma) (1 μM) and lucigenin (Sigma) (20 μM) were added, and chemiluminescence was determined using a Fluoroskan Ascent FL (Thermo*^®^*) in an out-of-coincidence mode.

### Determination of intracellular ROS generation

The H_2_O_2_ levels were evaluated using the dichlorofluorescin diacetate (DCFH-DA) method. Following treatment, 10 μM DCFH-DA was added to cells for 45 min at 37°C. The cells were then harvested and the fluorescence was determined using a Fluoroskan Ascent FL platereader with wavelengths of 485 nm (excitation) and 530 nm (emission).

### Chemicals and fluorescent probes for studying ROS generation

The fluorescent dyes were obtained from Molecular Probes (Eugene, Oreg., USA). With regards to loading conditions for the fluorescent probes, the mitochondrial membrane potential dye Tetramethyl rhodamine ethyl ester (TMRM) was used at 100 nM, while the ROS probe 6-carboxy-2′,7′-dichlorodihydrofluorescein diacetate (DCF) was used at 500 nM. Cells were incubated with the fluorescent probes for 20–30 min at room temperature, and then rinsed with PBS-buffered saline solution. Subsequently, cells were mounted on a cell chamber, and visualized using a Leica confocal microscope (model TCSNT) with filters specific for DCF (excitation 450–490 nm, emission 515–565 nm) and TMRM (excitation 540–550 nm, emission 590–600 nm).

### Electrophoretic mobility-shift assay (EMSA)

The procedures for EMSA has been described previously [33, 58, 60]. Nuclear proteins were isolated with NE-PER reagent (Pierce, Rockford, IL, USA) in accordance with the manufacturer's instructions. The Light Shift chemiluminescence EMSA kit (Pierce) was used to determine the NF-κB binding activity of nuclear proteins. The sequences of the double-stranded EMSA probes were 5′-AGTTGAGGGGACTTTCCCAGGC-3′ and 3′-TCAACTCCCCTGAAAGGGT CCG-5′ for NF-κB.

### Immunofluorescence staining

Growth-arrested A549 and H1299 cells were pretreatedwith or without inhibitorsfor 1 h, and then treated with 10 μM artocarpin. Cells were washed twice with ice-cold PBS, then fixed and incubated with an anti-p65 antibody [61]. Images werevisualizedby fluorescence microscopy (Zeiss, Axiovert 200 M).

### Mice tumor xenograft study

Six weeks old male SCID mice [BALB/cA-nu (nu/nu)] were obtained from the National Science Council Animal Center (Taipei, Taiwan) and kept in a pathogen-free environment. A549 or H1299 cells (1 × 10*^6^* cells in 200 μl normal saline) were injected subcutaneously into the flanks of mice, and tumors were allowed to grow for 14 days, reaching approximately 100 mm*^3^* in size. The mice (10 mice/group) were then treated with vehicle or 1 mg/kg artocarpin daily for 21 days. The tumor volume was determined twice a week using a caliper, according to the formula V = (LW2) p/6: where V = volume (mm*^3^*), L = biggest diameter (mm), W = smallest diameter (mm). Animal studies adhered to institutional guidelines and were approved by the Animal Care Committee of Shin-Kong Wu Ho-Su Memorial Hospital.

## SUPPLEMENTARY MATERIALS AND METHODS AND FIGURES


