# Correction: Artocarpin, an isoprenyl flavonoid, induces p53-dependent or independent apoptosis via ROS-mediated MAPKs and Akt activation in non-small cell lung cancer cells

**DOI:** 10.18632/oncotarget.26990

**Published:** 2019-05-21

**Authors:** Ming-Horng Tsai, Ju-Fang Liu, Yao-Chang Chiang, Stephen Chu-Sung Hu, Lee-Fen Hsu, Yu-Ching Lin, Zih-Chan Lin, Hui-Chun Lee, Mei-Chuan Chen, Chieh-Liang Huang, Chiang-Wen Lee

**Affiliations:** ^1^ Department of Pediatrics, Division of Neonatology and Pediatric Hematology/Oncology, Chang Gung Memorial Hospital, Yunlin, Taiwan; ^2^ Central Laboratory, Shin-Kong Wu Ho-Su Memorial Hospital, Taipei, Taiwan; ^3^ Center for Drug Abuse and Addiction, China Medical University Hospital, China Medical University, Taichung, Taiwan; ^4^ Department of Nursing, Division of Basic Medical Sciences, Chang Gung University of Science and Technology, Chia-Yi, Taiwan; ^5^ Department of Dermatology, College of Medicine, Kaohsiung Medical University, Kaohsiung, Taiwan; ^6^ Department of Dermatology, Kaohsiung Medical University Hospital, Kaohsiung, Taiwan; ^7^ Department of Respiratory Care, Chang Gung University of Science and Technology, Chiayi Campus, Chiayi, Taiwan; ^8^ Division of Pulmonary and Critical Care Medicine, Chang Gung Memorial Hospital, Chiayi, Taiwan; ^9^ Department of Respiratory Care, Chang Gung University, Taoyuan, Taiwan; ^10^ Graduate Institute of Biomedical Sciences, Chang Gung University, Taoyuan, Taiwan; ^11^ Program for the Clinical Drug Discovery from Botanical Herbs, College of Pharmacy, Taipei Medical University, Taipei, Taiwan; ^12^ Chronic Diseases and Health Promotion Research Center, Chang Gung University of Science and Technology, Chia-Yi, Taiwan; ^13^ Research Center for Industry of Human Ecology and Research Center for Chinese Herbal Medicine, College of Human Ecology, Chang Gung University of Science and Technology, Taoyuan, Taiwan; ^14^ Department of Rehabilitation, Chang Gung Memorial Hospital, Chia-Yi 61363, Taiwan

**This article has been corrected:** The new affiliation no.14 is given below:

**Chiang-Wen Lee**^14^

^14^Department of Rehabilitation, Chang Gung Memorial Hospital, Chia-Yi 61363, Taiwan

Original article: Oncotarget. 2017; 8:28342–28358. 28342-28358 . https://doi.org/10.18632/oncotarget.16058

