# Supplementary material for: Artocarpin, an isoprenyl flavonoid, induces p53-dependent or independent apoptosis via ROS-mediated MAPKs and Akt activation in non-small cell lung cancer cells
Source: Oncotarget. 2017 Mar 9;8(17):28342–58. doi: 10.18632/oncotarget.16058 (PMC5438654; doi:10.18632/oncotarget.16058)
Supplement: Supplementary file 1 [file oncotarget-08-28342-s001.pdf]

Artocarpin, an isoprenyl flavonoid, induces p53-dependent or independent apoptosis via ROS-mediated MAPKs and Akt activation in non-small cell lung cancer cells

Supplementary Materials

Reagents

MitoTempo (a specific scavenger for mitochondrial superoxide anions) was obtained from Cayman (Ann Arbor, MI, USA). MitoSOX Red mitochondrial superoxide indicator was purchased from Molecular Probes, Eugene, OR, USA. Caspase-3, -7, and -9 colorimetric assay kits were obtained from R&D Systems (Minneapolis, MN, USA).

Determination of mitochondrial ROS

A549 and H1299 cells were washed in warm HBSS, followed by incubation in HBSS or cell medium containing 5 μM MitoSOX Red mitochondrial superoxide indicator at 37°C for half an hour. Subsequently, fresh medium was added, cells were pre-incubated with or without inhibitor for 1 hr and then treated with artocarpin for 24 hr. After washing twice with PBS, cells were harvested with trypsin-EDTA, and the cellular fluorescence level was evaluated by flow cytometry at wavelengths of 510 nm (excitation) and 580 nm (emission).

Caspase activity determinations

The activities of caspases in cell lysates were evaluated using the caspase-3, -7, and -9 colorimetric assay kits in accordance with the manufacturer's protocols. Following treatment with artocarpin with or without inhibitors, A549 cells were lysed in lysis buffer. Specific substrates for caspase-3, -7, and -9 (Ac-DEVD-pNA and Ac-LEHD-pNA) were added to the cell lysates (50 μg proteins) at 37°C for one hour. The absorbance was then evaluated with a plate reader at a wavelength of 405 nm.

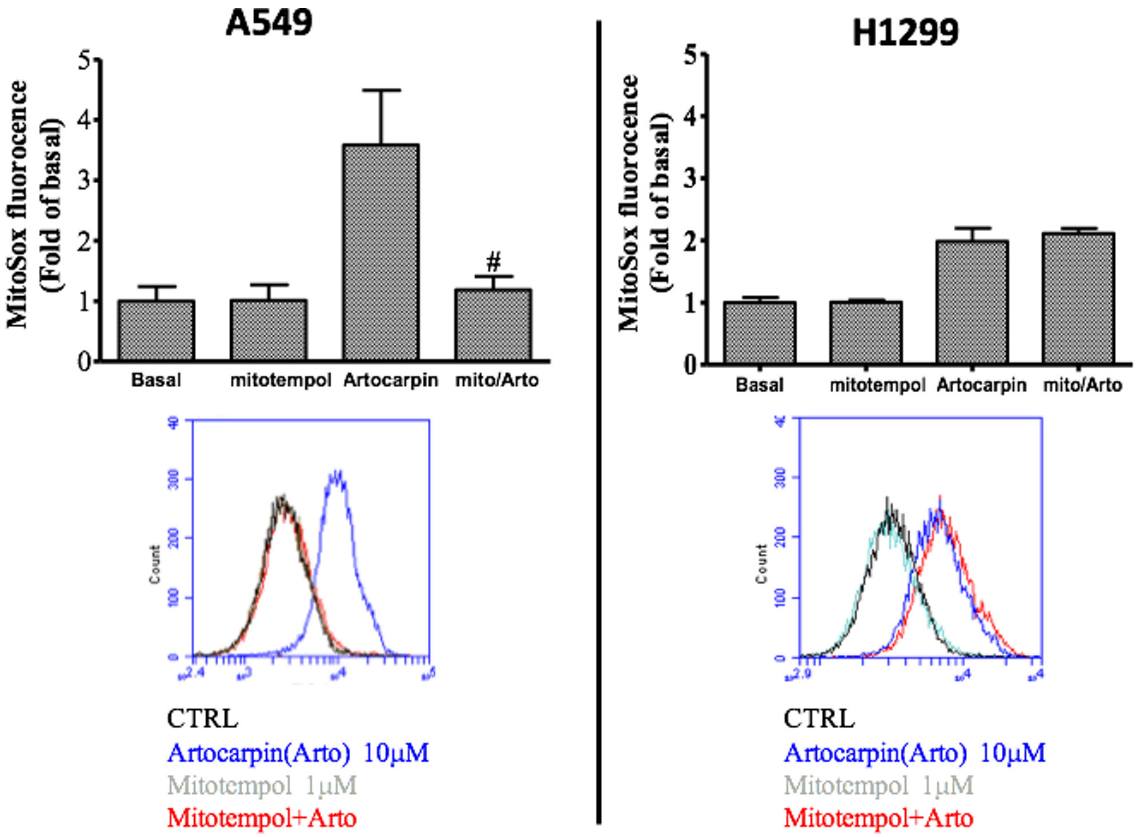

Supplementary Figure 1: Effect of mitoTEMPO on artocarpin-induced long-term production of mitochondrial ROS in A549 and H1299 cells. (**A**) Confluent cells were labeled with MitoSox (5 μM) and then pre-incubated with or without mitoTEMPO (1 μM). After incubation for 1 h, cells were treated with artocarpin (10 μM) for 24 h. The mitochondrial ROS production was measured using flow cytometry. Results are representative of three independent experiments.

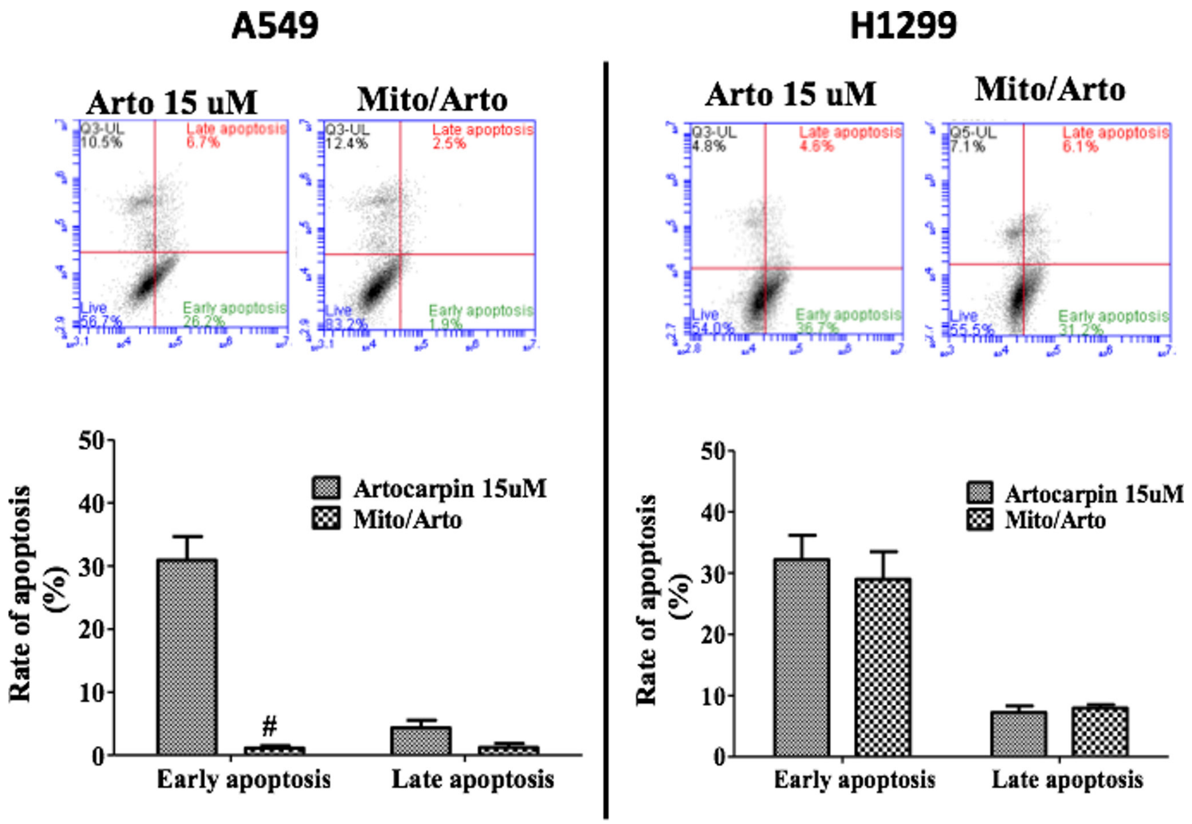

Supplementary Figure 2: Effect of mitoTEMPO on artocarpin-induced apoptosis in A549 and H1299 cells. (**A**) Confluent cells were labeled with Annexin-V and propidium iodide and then pre-incubated with or without mitoTEMPO (1 µM). After incubation for 1 h, cells were treated with artocarpin (15 µM) for 24 h. The rate of apoptosis was measured using flow cytometry. Results are representative of three independent experiments.

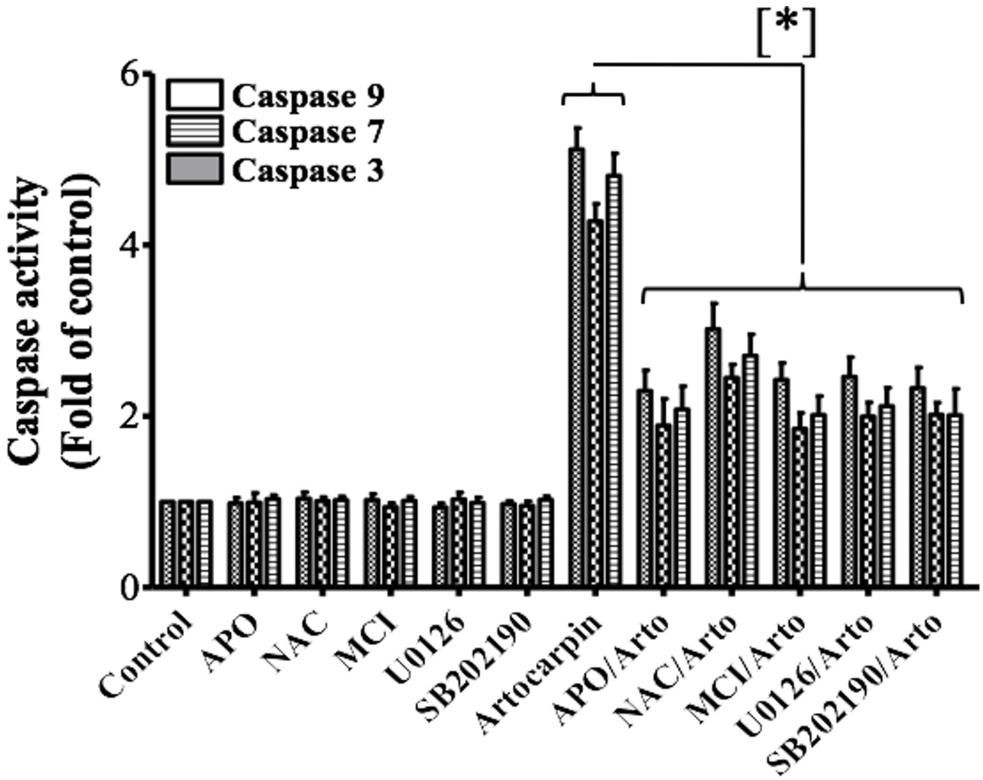

Supplementary Figure 3: Artocarpin induced apoptosis of A549 cells through activation of caspases. Confluent cells were pre-incubated with or without inhibitors of ERK1/2 (U0126 10 μM), p38 (SB202190 10 μM), NOX (APO 1 mM) and MCI-186 (antioxidant 10 μM). After incubation for 1 h, cells were treated with artocarpin (10 μM) for 24 h. The activities of caspases were analyzed by using caspase-3, -7, and -9 colorimetric assay kits. Results are representative of three independent experiments.
